# Input from multiple stakeholder levels prioritizes targets for improving implementation of an exercise intervention for rural women cancer survivors

**DOI:** 10.1186/s43058-020-00061-1

**Published:** 2020-11-04

**Authors:** Haiyan Qu, Richard Shewchuk, Xuejun Hu, Ana A. Baumann, Michelle Y. Martin, Maria Pisu, Robert A. Oster, Laura Q. Rogers

**Affiliations:** 1 University of Alabama at Birmingham, 1716 9th Ave S, Birmingham, AL 35294 USA; 2 Washington University in St. Louis, 1 Brookings Dr, St. Louis, MO 63130 USA; 3 Department of Preventive Medicine and Center for Innovation in Health Equity Research, University of Tennessee Health Science Center, 910 Madison Avenue, Memphis, TN 38163 USA; 4 School of Medicine, Division of Preventive Medicine, O’Neal Comprehensive Cancer Center, University of Alabama at Birmingham, 1720 2nd Ave S, MT 636, Birmingham, AL 35294-4410 USA; 5 School of Medicine, Division of Preventive Medicine, O’Neal Comprehensive Cancer Center, University of Alabama at Birmingham, 1720 2nd Ave S, MT 642, Birmingham, AL 35294-4410 USA; 6 School of Medicine, Division of Preventive Medicine, University of Alabama at Birmingham, 1720 2nd Ave S, MT 614, Birmingham, AL 35294-4410 USA

**Keywords:** Oncology, Physical activity, Qualitative, Nominal group technique, Implementation, Survivorship, Health promotion

## Abstract

**Background:**

Although evidence-based interventions for increasing exercise among cancer survivors (CSs) exist, little is known about factors (e.g., implementation facilitators) that increase effectiveness and reach of such interventions, especially in rural settings. Such factors can be used to design implementation strategies. Hence, our study purpose was to (1) obtain multilevel perspectives on improving participation in and implementation of a multicomponent exercise behavior change intervention for rural women CSs and (2) identify factors important for understanding the context using the Consolidated Framework for Implementation Research (CFIR) for comparison across three levels (CSs, potential interventionists, community/organizational stakeholders).

**Methods:**

We conducted three nominal group technique meetings with rural women CSs, three with community/organizational stakeholders, and one with potential interventionists. During each meeting, participants were asked to respond silently to one question asking what would make a multicomponent exercise intervention doable from intervention participation (CSs) or implementation (potential interventionists, stakeholders) perspectives. Responses were shared, discussed to clarify meaning, and prioritized by group vote. Data was deductively coded using CFIR.

**Results:**

Mean age of CSs (*n* = 19) was 61.8 ± 11.1 years, community stakeholders (*n* = 16) was 45.9 ± 8.1 years, and potential interventionists (*n* = 7) was 41.7 ± 15.2 years. There was considerable consensus among CSs, potential interventionists, and stakeholders in terms of CFIR domains and constructs, e.g., “Design quality and packaging” (Innovation Characteristics), “Patients needs and resources” (Outer Setting), “Available resources” (Inner Setting), and “Engaging” (Process). However, participant-specific CFIR domains and constructs were also observed, e.g., CSs endorsed “Knowledge and beliefs about the intervention,” “Individual stage of change,” and “Self-efficacy” (Characteristics of Individuals); potential interventionists valued “Tension for change” (Inner Setting) and “Innovation participants” and “Key stakeholder” (Process); stakeholders emphasized “Goals and feedback” and “Network and communication” (Inner Setting), and “Planning” (Process). How the three participant levels conceptualized the CFIR constructs demonstrated both similarities and differences.

**Conclusions:**

Multilevel input yielded diversity in type, relative priority, and conceptualization of implementation facilitators suggesting foci for future implementation strategy development and testing. Findings also reinforced the importance of multilevel implementation strategies for increasing exercise in an underserved, at-risk population.

Contributions to the literature

- Exercise can reduce mortality and morbidity after cancer. Hence, identifying implementation strategies is crucial for advancing the implementation science needed to translate exercise programs for cancer survivors into practice.
- Our findings identify and prioritize factors to be considered when designing strategies for successfully implementing an exercise program for rural cancer survivors from the perspective of three different types of stakeholders.
- Using the Consolidated Framework for Implementation Research (CFIR), our findings suggest differences in stakeholder perspectives that may guide strategy development.
- Our findings focus on an underserved population that suffers disparities related to health and physical inactivity.

## Background

With technical advances in cancer diagnosis and treatment, cancer survivors (CSs) can live longer [1–3], and the number of female CSs is estimated to reach over 10 million in the USA by 2026 [1]. Strong evidence has been found that exercise positively influences quality of life in women living with a history of cancer [4–7], such as beneficial effects on physical function [8–10], cognitive function [8, 11], fatigue [11–14], anxiety [8, 12–14], depression [9, 12, 14], insomnia [11–13], sexuality [11, 15], and cardiorespiratory fitness [8]. Also, a large body of research supports that exercise is associated with reduced cancer mortality risk in CSs [16–21]. For instance, women with a history of breast cancer who participate in exercise were at a 44% lower risk of mortality compared to those who did not engage in exercise [20]. Despite increasing awareness that exercise has critical implications for the CS and should be integrated into the survivorship care plan [22], many challenges remain and most women CSs are insufficiently physically active [23–26]. This is especially true for CSs in rural areas, an underserved population that is at greater risk for physical inactivity [27]. Furthermore, multiple evidence-based exercise promotion interventions exist yet few are successfully translated to broader, non-research settings and little data exist describing potential implementation construct targets for which implementation strategies should be designed and tested, especially in rural settings [28]. It is vital, therefore, to identify factors that facilitate implementation of exercise programs for rural women CSs, an at-risk population with unique characteristics and needs.

The Consolidated Framework for Implementation Research (CFIR), a comprehensive taxonomy that unifies and consolidates the array of constructs influencing implementation, has been used across a wide variety of study objectives and settings [29, 30]. Its standardized constructs related to implementation effectiveness of innovative programs are organized into the five major domains of Innovation Characteristics, Outer Setting, Inner Setting, Characteristics of Individuals, and Process [31]. Those standardized constructs provide an approach to articulate the determinants of implementation success and a guide to identify factors that are most important to implementation [30]. A body of qualitative and mixed-method studies have adopted CFIR to develop interview guides [32], form codebooks [33], analyze and interpret data [34], and report implementation-related findings [29]. These reports provide strong support for incorporating CFIR constructs when identifying multilevel factors that facilitate or hinder implementation, including the implementation of exercise programs for rural CSs [33–37]. However, a knowledge gap remains in that it is not known which CFIR constructs are endorsed by CSs vs. interventionists vs. stakeholders when considering implementation of an exercise program for CSs living in rural settings.

Given the need for exercise promotion among women CSs living in rural settings and the usefulness of CFIR, our study aims were as follows: (1) obtain multilevel perspectives (CSs, potential interventionists, community/organizational stakeholders) on improving participation in and implementation of a multicomponent exercise behavior change intervention for rural women CSs and (2) identify factors important for understanding the context using the CFIR for comparison across three levels (CSs, potential interventionists, community/organizational stakeholders). The intervention being translated is a 3-month program based on the social cognitive theory. It includes 12 supervised exercise sessions over a 6-week period that are tapered to entirely unsupervised exercise sessions during the final 6 weeks. Additional exercise education is provided by update exercise counseling sessions every 2 weeks during the home-based phase. Additional behavior change support is provided during six group discussions over the first 8 weeks that promote the development of a personalized behavioral modification plan [38].

## Methods

### Participants

To identify potential factors that facilitate the implementation of a multicomponent exercise behavior change intervention [38] by non-research staff in a rural, community-based health care organization, we obtained input from rural women CSs, potential interventionists, and community stakeholders in a rural Southeastern US county prior to intervention implementation. Participants lived or worked in or adjacent to the research study county, which was classified as rural based on Rural-Urban Continuum Code (RUCC) codes (completely rural or < 2500 urban population) [39]. Other inclusion criteria included age ≥ 18 years, intact hearing, English speaking, no history of dementia or organic brain syndrome, and no significant medical (e.g., infectious disease preventing group participation), psychological (e.g., cognitively unable to give informed consent), or social (e.g., overwhelmed by social stressors) characteristics that would interfere with the ability to fully participate. Potential interventionists were individuals who were qualified to implement the exercise sessions/counseling, coordinate intervention activities, and/or lead discussion group components of the program (e.g., fitness specialists or instructors, administrative staff, gym managers, patient navigators, and health educators). Stakeholders were community or organizational individuals who were qualified to facilitate and/or support program implementation activities (e.g., hospital administrators, health care professionals [nurse or physician], community organization representatives, social workers, or occupational therapists). Other inclusion criteria specific to CSs included women with a history of any cancer type (excluding skin cancer other than melanoma), stage, and number of years since diagnosis, post-primary cancer treatment, ambulatory without assistance, no contraindication to moderate intensity exercise, and able to obtain physician clearance for participation in moderate intensity exercise. Our CS study criteria were broad and no CSs were excluded. Two CSs refused participation (transportation limitations and lack of time due to family obligations) and one CS was lost to follow-up between screening and enrollment. All participants were recruited using local news ads, referrals from the local cancer center, and meetings with the cancer center leadership and project champion. The project was approved by the local institutional review boards and all participants provided informed consent before initiating study activities.

### Nominal group technique

Participants took part in nominal group technique (NGT) sessions. NGT is a well-established multi-step facilitated group meeting used with informant panels to elicit and prioritize responses to a specific question [40]. The details of the NGT methods have been described elsewhere [41–44]. Less structured group approaches (e.g., focus groups) can limit breadth of responses such as occurs when a small subset of focus group participants dominate the discussion. The highly structured NGT meeting minimizes such process loss and elicits a greater volume of novel and high-quality responses [45–47]. The NGT provides concise recorded documentation of participants’ verbatim responses, eliminates a potential source of investigator induced interpretive bias, and promotes even rates of participation. Moreover, NGT equally weights the input from all participants’ anonymously ranked responses that are assumed to reflect the collective views held by group participants [41, 48].

We prepared several candidate questions intended to elicit facilitators of participation in or implementation of an intervention that promotes exercise among women CSs in rural areas. After reviewing question accuracy and clarity, the final questions selected for the NGT panel meetings were (1) CSs = “What sorts of things would make this intervention doable for cancer survivors around here?” and (2) interventionists and stakeholders *=* “What sorts of things would make delivering this program doable in this area?”.

At the beginning of each NGT meeting, we briefly described the planned intervention and purpose of the meeting to participants. They were asked to silently generate and write their responses to the NGT question. Then, participants presented their responses to the group using a round-robin format. The responses were recorded on a flipchart. After the responses were exhausted, participants from each panel were given an opportunity to briefly discuss responses for clarification (not evaluation), to ensure that every response was understood from a common perspective. During these discussion phases, some responses were elaborated and a small number of responses were added. The final phase of each meeting consisted of a structured prioritization exercise that involved having each participant anonymously select from the group list what they individually perceived as the three most important things/factors from the group list and were not limited to selecting things/factors that they themselves nominated. Each participant was given a total of six votes and was asked to assign votes to the three things/factors based on perceived importance (i.e., most important received 3 votes, next most important received 2 votes, and third most important received 1 vote). The duration of each meeting varied, but on average was approximately 90 min. Weighted vote tally for a specific item was calculated by summing the weighted votes received (e.g., an item receiving a vote from three participants, all giving a “1” for third most important would have a weighted vote tally of 3; an item receiving a vote from only one participant who voted a “3” for most important would also receive weighted vote tally of 3). All weighted vote tallies were summed for a total for the group (i.e., the number of group members multiplied by 6). The total vote amount was used as the denominator to calculate the percentage of total votes received by each factor; the percentage represents the consensus or agreement across participants in each NGT group.

### Analysis

Descriptive statistics were conducted for the participant characteristics. Frequencies and percentages were calculated for categorical variables (i.e., gender, race, marital status, income, and cancer types). Means and standard deviations were calculated for continuous variables (i.e., age, education level, years of diagnosis, and distance from home). Fisher’s exact test was used for the analysis of the categorical variables due to small frequencies in the contingency tables. Analysis of variance, followed by the Tukey-Kramer multiple comparisons test for pairwise differences, was used for the analysis of the continuous variables. Statistical tests were two-sided and were performed using a significance level of 5%. SAS, Version 9.4 (SAS Institute, Cary, NC), and Tableau, Version 2018.3.2 (Seattle, WA Tableau) were used for the statistical and visualization analyses.

The responses from each NGT meeting were entered and listed in tables. The individual rank orderings from each NGT meeting were aggregated across participants to tabulate a group level result. To broaden data utility, suggested facilitators were coded into the implementation science construct with the goal of using these constructs to guide the design of potentially useful implementation strategies. As such, the prioritized NGT responses were further coded based on CFIR domains and constructs for different participant types using the CFIR codebook (http://www.cfirguide.org/tools.html) by two research staff individually. Coding was iteratively discussed to reconcile coder differences. The percent of times a theme (i.e., CFIR code) was identified and calculated for CSs, interventionists, and stakeholders separately.

## Results

### Participant characteristics

Participant characteristics are displayed in Table 1. The mean age of CSs (*n* = 19) was 61.8 ± 11.1 years (range 44 to 83 years). Nearly half of CSs reported a history of breast cancer (47.4%) with the remaining reporting bladder, lung, ovarian, uterine, melanoma, or leukemia; 57.9% were early stage; 78.9% were White; 42.1% reported annual income under $50,000; and the mean education level was 15.1 ± 2.7 years. The mean age of interventionists (*n* = 7) was 41.7 ± 15.2 years and stakeholders (*n* = 16) was 45.9 ± 8.1 years.

**Table 1:** Characteristics and Consolidated Framework for Implementation Research (CFIR) domains by participant type (*N* = 42)

		Cancer survivor (*n* = 19)	Potential interventionist (*n* = 7)	Stake-holder (*n* = 16)	Total (*N* = 42)	*p* value
		*N* (%)	*N* (%)	*N* (%)	*N* (%)	
Gender	Male	0 (0)	2 (28.6)	4 (25.0)	6 (14.3)	0.03
	Female	19 (100.0)	5 (71.4)	12 (75.0)	36 (85.7)	
Race	White	15 (78.9)	7 (100.0)	15 (93.8)	37 (88.1)	0.37
	Black	4 (21.1)	0 (0)	1 (6.3)	5 (11.9)	
Income	< $50,000	8 (42.1)	1 (14.3)	0 (0)	9 (21.4)	0.01
	≥ $50,000	11 (57.9)	6 (85.7)	16 (100.0)	33 (78.6)	
Married	Yes	13 (68.4)	6 (85.7)	14 (87.5)	33 (78.6)	0.47
	No	6 (31.6)	1 (14.3)	2 (12.5)	9 (21.4)	
Cancer type^a^	Breast	9 (47.4)	–	–	9 (47.4)	–
	Other	10 (52.6)	–	–	10 (52.6)	
Early cancer stage^a^	Yes	11 (57.9)	–	–	11 (57.9)	–
	No	8 (42.1)			8 (42.1)	
		Mean (SD)	Mean (SD)	Mean (SD)	Mean (SD)	
Age, years		61.8 (11.1)	41.7 (15.2)	45.9 (8.1)	52.4 (13.7)	< 0.001^b^
Education, years		15.1 (2.7)	16.4 (0.8)	17.3 (1.9)	16.2 (1.4)	0.019^c^
Years since diagnosis^a,d^		2.8 (1.9)	–	–	2.8 (1.9)	–
Miles from home to anticipated intervention site		20.3 (17.9)	27 (20)	29.0 (11.5)	24.7 (16.3)	0.27
CFIR domain		*N* (%)^e^	*N* (%)	*N* (%)	*N* (%)	
Innovation Characteristics		36 (37.5)	8 (25.0)	32 (33.7)	76 (34.1)	0.57
Outer Setting		9 (9.4)	4 (12.5)	9 (9.5)	22 (9.9)	
Inner Setting		19 (19.8)	10 (31.3)	22 (23.2)	51 (22.9)	
Characteristics of Individuals		13 (13.5)	1 (3.1)	4 (4.2)	18 (8.1)	
Process		19 (19.8)	9 (28.1)	28 (29.5)	56 (25.1)	
Total		96 (100.0)	32 (100.0)	95 (100.0)	223 (100.0)	

^a^Applies only to cancer survivors

^b^The mean age of the cancer survivors is significantly greater than the mean age of the interventionists and mean age of the stakeholders

^c^The mean education level of the stakeholders is significantly greater than the mean education level of the cancer survivors

^d^*n* = 18

^e^Number (%) of times CFIR domain coded; % = (Number of each CFIR domain [N]/Total number of all 5 CFIR domains) × 100

Statistically significant associations existed between income and group (*p* = 0.01), with greater income in the interventionist and stakeholder groups when compared to CSs. Given our focus on women CS, male representation was only present in the interventionist and stakeholder groups. There were also statistically significant differences between the mean age of the groups (*p* < 0.001) and mean education level of the groups (*p* = 0.019), with the mean age of the CSs being greater than that of the interventionists and that of the stakeholders, and the mean education level of the stakeholders being greater than that of the CSs.

### NGT results

A total of 223 factors anticipated to influence implementation success were generated from 7 NGT meetings, of which 79 factors were selected and prioritized. All participants in each group endorsed a prioritized list and verbally confirmed that it accurately reflected the group’s discussion. The prioritized list of NGT responses was reported for each participant group in Tables 2, 3, and 4.

**Table 2:** Cancer survivors (*N* = 19) prioritized perceived implementation facilitators during nominal group technique (NGT) meetings

Response	*N*	Votes assigned	Sum	% of votes	CFIR domain: *CFIR constructs*
**CS NGT group 1 (*n*** **= 6)**	**18**		**36**	**100**	
Commitment	3	3,3,3	9	25.00	CI: *Knowledge and beliefs about the intervention*
Flexibility (time, exercise type, level, schedule)	4	3,3,1,1	8	22.22	IC: *Design quality and packaging*
Access to exercise specialist (e.g., learn exercise knowledge, how to use exercise machines)	2	2,1	3	8.33	IS: *Readiness for Implementation; Available resources*
Fee free for class, membership, and exercise specialist	1	3	3	8.33	IC: *Cost*
Provide exercise structure (e.g., amount of time, right ways, and how)	1	2	2	5.56	IS: *Readiness for Implementation; Access to knowledge and information; Available resources*
Convenient (e.g., parking, close enough to where they live)	1	2	2	5.56	IS: *Readiness for Implementation; Access to knowledge and information; Available resources*
Provide nutrition information/class (e.g., Print, 5 - minute video online, show/recipe)	1	2	2	5.56	IS: *Readiness for Implementation; Access to knowledge & information*
Overcome fears/resistance (e.g., fear of exercise, fear of injury)	1	2	2	5.56	IC: *Self-efficacy; Design quality and packaging*
Time/length of exercise (e.g., 150 min/week; daily time)	1	2	2	5.56	IC: *Self-efficacy; Design quality and packaging*
Get support from family, friends, peers, church, and facilities here	1	1	1	2.78	P: *Engaging; External change agents*
Having a buddy to go with	1	1	1	2.78	OS: *Patient needs and resources*
Technology (e.g. Fitbit; online program/show)	1	1	1	2.78	IC: *Design quality and packaging*
**CS NGT group 2 (*n*** **= 8)**	**24**		**48**	**100**	
Effective exercise (advanced, beginner)	8	3,3,3,3,3,2,2,1	20	41.67	IC: *Design quality and packaging*
Goal setting (writing down goals, better chances of reaching them; personal goals; on own and talking with specialist [motivating to do on own])	2	3,2	5	10.42	IC: *Design quality and packaging*
Flexible schedule/time for working/non-working survivors	2	3,1	4	8.33	IC: *Design quality and packaging*
Should be fun (IMPORTANT! Lack of participation/enthusiasm without it )	1	3	3	6.25	IC: *Design quality and packaging*
Central location/easy access	2	1,1	2	4.17	IS: *Readiness for Implementation*
Workout partner	2	1,1	2	4.17	OS: *Patient needs and resources*
Leadership/administration team (consistency of how program ran; not too many moving parts)	1	2	2	4.17	IS: *Readiness for Implementation; Leadership engagement; Culture*
Exercise specialist focused on cancer survivors	1	2	2	4.17	IS: *Readiness for Implementation; Leadership engagement; Culture*
Encouragement throughout the program	1	2	2	4.17	IS: *Readiness for Implementation; Leadership engagement; Culture*
Mental health-focused groups (group sessions discuss/share struggles; more structured; led by mental health counselor; get support; discuss success; sharing ideas of past successes)	1	2	2	4.17	IC: *Design quality and packaging*
Way to measure progress	1	2	2	4.17	IC: *Design quality and packaging*
Peer support (e.g., work out partner/buddy)	1	1	1	2.08	OS: *Patient needs and resources*
Description of possible results of exercise types (provide information; e.g., aerobics helps lung function; individualized)	1	1	1	2.08	CI: *Knowledge and beliefs about the intervention*
**CS NGT group 3 (*n*** **= 5)**	**15**		**30**	**100**	
Convenient location–distance (save travel time, easier to drive)	3	3,3,2	8	26.67	IS: *Readiness for Implementation; Available resources*
Inexpensive cost, affordability	2	3,1	4	13.33	*IC: Cost*
Facility with various equipment (exercise different body parts, not to get bored)	2	2,2	4	13.33	IS: *Readiness for Implementation; Available resources*
Many forms of advertising	2	2,1	3	10.00	P: *Engaging*
Knowledge of self-care	1	3	3	10.00	OS: *Patient needs and resources*
Staff and participants show caring	1	3	3	10.00	*IS: Culture*
Group exercise sessions	2	1,1	2	6.67	IC: *Design quality and packaging*
Family support	1	2	2	6.67	P: *Engaging; External change agents*
Exercise partner—accountability	1	1	1	3.33	*CI: Other personal attributes*
**Grand total (*N*** **= 19 CS participants)**	**57**		**114**		

Legend: Prioritization of perceived things that would make a multicomponent exercise intervention doable for rural cancer survivors (CS) by 19 CSs, i.e., the most important things out of the 96 total suggestions generated

*NGT* nominal group technique, *% of votes* (Number of votes for each response [N]/Sum of votes from each NGT group[Sum]) × 100, *CFIR* Consolidated Framework for Implementation Research, *IC* Innovation Characteristics, *OS* Outer Setting, *IN* Inner Setting, *CI* Characteristics of Individuals, *P* Process

**Table 3:** Interventionists (*N* = 7) prioritized perceived implementation facilitators during nominal group technique (NGT) meetings

Response	*N*	Votes assigned	Sum	% of votes	CFIR domain: *CFIR constructs*
**Interventionist NGT group (*N*** **= 7)**	**21**		**42**	**100**	
Financial support (i.e., corporate support, endowments, grants)	4	3,3,2,1	9	21.43	IC: *Cost*
Committed exercise specialist (e.g., competent, energetic, qualified, making the most of time)	3	2,2,1	5	11.90	P: *Engaging, Formally appointed internal implementation leaders*
Set realistic/attainable goals for participants (e.g., short-term goals easy to accomplish)	2	3,1	4	9.52	IC: *Design quality and packaging*
Free transportation	2	2,1	3	7.14	IS: *Readiness for Implementation; Available resources; Leadership engagement*
Medical oversight and staff oversight	1	3	3	7.14	IS: *Readiness for Implementation; Available resources; Leadership engagement*
Ensure demand for program	1	3	3	7.14	IS: *Implementation Climate; Tension for change*
Physician clearance	1	3	3	7.14	P: *Engaging; External change agents; Key stakeholders*
Educate referral source	1	3	3	7.14	P: *Engaging; External change agents; Key stakeholders*
Community support (e.g., awareness of need, availability of locations, use of gym)	1	2	2	4.76	IS: *Readiness for Implementation; Available resources*
Research to support program (e.g., awareness of benefits, success stories, testimonials, validation)	1	2	2	4.76	IC: *Evidence strength and quality*
Integrate program with non-participants and community (e.g., family, spouse education, support)	1	2	2	4.76	OS: *Cosmopolitanism*
Trainers understand participants (e.g., emotion/mental, past history of patients, limitations of cancers specific, type of cancer)	1	1	1	2.38	OS: *Patient needs and resources*
Recruit participants with potential for success	1	1	1	2.38	P: *Engaging; Innovation participants*
Exercise specialists chart progress (e.g., physiologic parameters, heart rate, distance, etc.)	1	1	1	2.38	IC: *Design quality and packaging*

Legend: Prioritization of perceived things that would make a multicomponent exercise intervention doable for a rural organization by seven interventionists, i.e., the most important things out of the 32 total suggestions generated

*NGT* nominal group technique, *% of votes* (Number of votes for each response [N]/Sum of votes from each NGT group[Sum]) × 100, *CFIR*: Consolidated Framework for Implementation Research, *IC*: Innovation Characteristics, *OS*: Outer Setting, *IN*: Inner Setting, *CI*: Characteristics of Individuals, *P*: Process

**Table 4:** Community/organizational stakeholders (*N* = 16) prioritized perceived implementation facilitators during nominal group technique (NGT) meetings

Response	*N*	Votes assigned	Sum	% of votes	CFIR domain: *CFIR constructs*
**Stakeholder NGT group 1 (*n*** **= 6)**	**18**		**36**	**100**	
Qualified trainer that can motivate CSs	4	3,3,3,2	11	30.56	IC: *Design quality and packaging*
A referral from doctors or nurse practitioners	2	3,2	5	13.89	P: *Engaging; External change agents*
Promotion and awareness (e.g., newspaper, marketing, oncologist office, church, radio station, hospital website)	2	2,2	4	11.11	P: *Engaging*
Grant money or other resources to fund program (e.g., pay for refreshment, trainer, donation, operational costs)	1	3	3	8.33	IC: *Cost*
Training for start-up, train trainers	1	3	3	8.33	IS: *Readiness for Implementation; Access to knowledge and information*
Buy-in from healthcare provider (trust doctors, nurse practitioners)	2	1,1	2	5.56	P: *Engaging; External change agents*
A good location/safe area, convenient/easy access	2	1,1	2	5.56	IS: *Readiness for Implementation; Available resources*
Financially feasible for participants	1	2	2	5.56	IC: *Cost*
Person-centered/individualized	1	2	2	5.56	IC: *Adaptability*
Transportation assistant, church, pick-up van, car pool	1	1	1	2.78	IS: *Readiness for Implementation; Available resources*
Flexible schedule, duration	1	1	1	2.78	IC: *Design quality and packaging*
**Stakeholder NGT group 2 (*n*** **= 5)**	**15**		**30**	**100**	
Convenience (e.g., time, location)	3	3,3,3	9	30.00	IC: *Design quality and packaging*
Good communication tools	2	2,2	4	13.33	IS: *Readiness for Implementation; Available resources*
Dedicated team (e.g., personalized, whole structure of program)	2	3,1	4	13.33	P: *Engaging*
Champion for program	1	3	3	10.00	P: *Engaging; Champions; External change agents*
Physician/nursing staff engagement	2	2,1	3	10.00	P: *Engaging; Champions; External change agents*
Accountability for CS and interventionist engagement	1	2	2	6.67	CI: *Other personal attributes*
Community awareness (people need to know it's available to participate)	1	2	2	6.67	P: *Engaging*
Use of incentives (e.g., T-shirt for CSs, incentives to interventionists)	1	1	1	3.33	P: *Engaging*
Providing weekly/daily feedback about exercise progress to CSs	1	1	1	3.33	P: *Reflecting and evaluating*
Use of UAB brand (e.g., experienced research team)	1	1	1	3.33	OS: *Cosmopolitanism*
**Stakeholder NGT group 3 (*n*** **= 5)**	**15**		**30**	**100**	
Physician buy-in (source of trust in program)	4	3,3,2,1	9	30.00	P: *Engaging; External change agents*
Transportation assistance for CSs (e.g., gas card)	3	3,2,2	7	23.33	IS: *Readiness for Implementation; Available resources*
Applying for funding to support staff (e.g., foundation, charitable)	1	3	3	10.00	IC: *Cost*
Pair participants with each other or other support person	1	3	3	10.00	OS: *Patient needs and resources*
Coordinator for entire program	1	2	2	6.67	P: *Engaging; Formally appointed internal implementation leaders*
Reward for staying the course (e.g., gym membership for CSs, incentives to interventionists)	1	2	2	6.67	P: *Engaging*
Advertisement (e.g., billboard; help understand program; help reach those out of treatment)	1	1	1	3.33	P: *Engaging*
Involvement of CSs (Previous CSs would be a great resource of support)	1	1	1	3.33	P: *Engaging; Champions*
Coordination with local gyms	1	1	1	3.33	IS: *Readiness for Implementation; Available resources*
Convenient hours (e.g., options for those who work or can't drive at night)	1	1	1	3.33	IC: *Design quality and packaging*
**Grand total (*N*** **= 16 stakeholder participants)**	**48**		**96**		

Legend: Prioritization of perceived things that would make a multicomponent exercise intervention doable for a rural organization by 16 community/organizational stakeholder participants, i.e., the most important things out of the 95 total suggestions generated

*NGT* nominal group technique, *CS* cancer survivor, *% of votes* (Number of votes for each response [N]/Sum of votes from each NGT group[Sum]) × 100, *CFIR* Consolidated Framework for Implementation Research, *IC* Innovation Characteristics, *OS* Outer Setting, *IN* Inner Setting, *CI* Characteristics of Individuals, *P* Process

#### CS NGT group 1 (*n* = 6)

Six CS participants in panel 1 generated 36 factors potentially influencing implementation, and they selected and prioritized 12. Three participants voted “CS commitment” as the most important factor (e.g., commitment to the exercise intervention program), accounting for 25.0% (9) of 36 total votes. Four participants assigned 8 votes to “Flexibility (i.e., time, exercise type, level, schedule),” accounting for 22.2% of total votes. Two participants assigned 3 votes to “Access to exercise specialist” (8.33%). One participant voted “Fee free for class, membership, and exercise specialist” (8.3%) as her most important. The top 4 factors for the CS panel 1 accounted for 63.9% of total votes (Table 2).

#### CS NGT group 2 (*n* = 8)

Thirteen of 42 responses were prioritized by CS panel 2. All participants in CS panel 2 assigned their votes to “Effective exercise” (for both beginner and advanced level) (e.g., different types and intensity levels of exercises help CS with different needs), accounting for 41.7% of total votes. Five of them voted “Effective exercise” as their most important factor and two as their 2^nd^ most important one. Two participants in CS panel 2 voted “Goal setting” (10.4%) and “Flexible schedule/time for working/non-working survivors” (8.3%). One CS voted “Should be fun” (6.3%) as the most important factor. The top 4 voted factors took up 66.7% of the total votes (Table 2).

#### CS NGT group 3 (*n* = 5)

Nine out of 18 responses were selected as important factors. Three participants in CS panel 3 endorsed “Convenient location” (26.7%) as either the most important or the 2^nd^ most important factor. “Inexpensive costs, affordability” (13.3%) and “Facility with various equipment” (13.3%) were ranked equally important factors. Two CSs voted “Many forms of advertising” as important (10.0%). One CS assigned “Knowledge of self-care” (10.0%) and the other ranked “Staff and participants show caring” (10.0%) as most important. The top 6 factors received 83.3% overall group votes (Table 2).

#### Interventionist NGT group (*n* = 7)

Among 32 factors generated by the interventionist panel, 14 were endorsed and ranked. Four interventionists assigned 9 votes to “Financial support” (21.4%). Three interventionists assigned 5 votes to “Committed exercise specialist (e.g., competent, energetic, qualified, making the most of time)” (11.9%). Four votes were assigned by two interventionists to “Set realistic/attainable goals for participants (e.g., short-term goals to accomplish)” (9.5%). “Free transportation” was selected by two participants (7.1%). “Ensure demand for program” (7.1%), “Medical oversight and staff oversight” (7.1%), “Physician clearance” (7.1%), and “Educate referral source” (7.1%) were each endorsed as most important by one interventionist. The top 8 factors received 78.6% total group votes (Table 3).

#### Stakeholder NGT group 1 (*n* = 6)

Stakeholder participants generated 26 factors, selected and ranked 11 of them. Four stakeholders assigned 11 votes to “Qualified trainer that can motivate cancer survivors” (30.6%). Three stakeholders assigned this factor as their most important and one stakeholder voted it as his/her 2^nd^ most important factor. Two stakeholders gave 5 votes to “A referral from doctors or nurse practitioners” (13.9%). “Promotion and awareness, e.g., newspaper, marketing, oncologist office, church, radio station, hospital website” received 4 votes from two panelists (11.1%). Two stakeholders ranked “Grant money or other resources to fund program, e.g., pay for refreshment, trainer, donation, operational costs” (8.3%) and “Training for start-up, train trainers” (8.3%) as most important, individually. The top 5 factors received 72.2% total votes (Table 4).

#### Stakeholder NGT group 2 (*n* = 5)

Participants in this panel generated 34 factors and ranked 10. Three stakeholders assigned their most important votes to “Convenience, e.g., time, location” (30.0%). “Good communication tools” (13.3%) and “Dedicated team, e.g., personalized, whole structure of program” (13.3%) received the 2^nd^ most important votes from two stakeholders, respectively. One stakeholder assigned “Champion for program” (10.0%) as his/her most important. “Physician/Nursing staff engagement” (10.0%) received 3 votes from two participants. The first 5 factors accounted for 76.7% of total group votes (Table 4).

#### Stakeholder NGT group 3 (*n* = 5)

This stakeholder group generated 35 responses and endorsed and prioritized 10. Four of 5 stakeholders assigned 9 votes to “Physician buy-in (source of trust in program)” (30.0%). Three stakeholders gave 7 votes to “Transportation assistance for cancer survivors (e.g., gas card)” (23.3%). “Applying for funding to support staff, e.g., foundational, charitable” (10.0%) and “Pair participants with each other or other support person” (10.0%) were ranked as the most important. The top 4 factors received 73.3% of total votes (Table 4).

### CFIR results

**Fig. 1:**
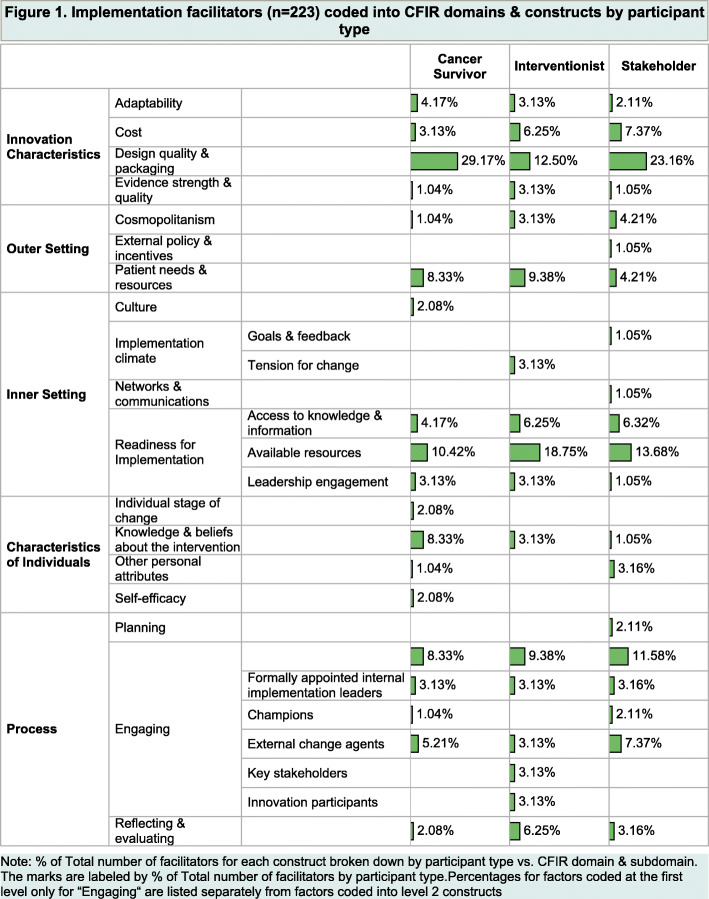
Implementation facilitators (*n* = 223) coded into CFIR domains and constructs by participant type

The CFIR domains and constructs coded from the 223 NGT responses are aggregately depicted in Table 1 and Fig. 1 by participant type. Results obtained from the Fisher’s exact test based on 10,000 Monte-Carlo simulations indicated that there was no statistically significant (*p* > 0.05) relationship between the five major CFIR domains and participant type (Table 1). Participant type comparisons across the constructs (subdomains) were not possible because of the small sample size within several subdomains. However, visualization of the subdomains for each participant group (Fig. 1) showed variability across participant type.

Nearly 30% of CS-generated responses related to the “Design quality and packaging” construct of the CFIR Innovation Characteristics domain vs. 12.5% interventionists and 23.2% stakeholders. The 2^nd^ largest CFIR construct for CSs was “Available resources” (10.4%) of Inner Setting domain. The 3^rd^ largest CFIR construct for CSs was “Patient needs and resources” (8.3%) of Outer Setting domain, “Knowledge and beliefs about the intervention” (8.3%) of Characteristics of Individuals domain, and “Engaging” (8.3% for factors coded at the first level only for “Engaging”) of Process domain (Fig. 1).

The two most frequent CFIR constructs for Interventionists were “Available resources” (18.8%) of Inner Setting domain, and “Design quality and packaging” (12.5%) of Innovation Characteristics domain. Responses fell equally (9.4%) into “Patient needs and resources” of Outer Setting domain and factors coded at the first level only for “Engaging” of Process domain. Similarly, responses were equal (6.3%) in “Cost” of Innovation Characteristics domain, “Access to knowledge and information” of Inner Setting domain, and “Reflecting and evaluation” of Process domain. The remaining responses fell equally into 10 sub-categories in five CFIR domains (Fig. 1).

The top three CFIR categories for stakeholders were “Design quality and packaging” (23.2%) of Innovation Characteristics domain, “Available resources” (13.7%) of Inner Setting domain, and first level only for “Engaging” (11.6%) of Process domain. The next three large categories were “Cost” (7.4%) of Innovation Characteristics domain, “External change agents” (7.4%) of Process domain, and “Access to knowledge and information” of Inner Setting domain (Fig. 1).

In summary, there was a considerable consensus among CSs, potential interventionists, and community/organizational stakeholders in terms of CFIR domains and constructs, e.g., “Design quality and packaging” of the Innovation Characteristics domain, “Patients needs and resources” of the Outer Setting, “Available resources” of the Inner Setting, and “Engaging” of the Process domain. However, participant-specific CFIR domains and constructs were also observed, e.g., CSs endorsed “Knowledge and beliefs about the intervention,” “Individual stage of change,” and “self-efficacy” of Characteristics of Individuals domain; interventionists valued “Tension for change” of Inner Setting domain, “Innovation participants” and “Key stakeholders” of Process domain; and stakeholders cared more about “Goals and feedback” and “Network and communication” of Inner Setting domain, and “Planning” of Process domain (Fig. 1).

Also relevant to group-specific findings, the data provided in Tables 2, 3, and 4 suggest similarities and differences between the three participant levels with regard to conceptualization of the constructs prioritized. This is exemplified by “Readiness” conceptualized by CSs as including (but not limited to) gaining personal knowledge regarding how to exercise while interventionists included factors such as insuring medical/staff oversight and stakeholders included training the exercise trainers. For “Engaging,” the three participant types included engaging with community partners and recruiting participants with advertising and/or incentives while CSs also mentioned engaging friends and family, interventionists and stakeholders mentioned engaging physicians for referrals, and stakeholders included engaging a program champion and coordinator. With regard to “Cost,” CSs focused on personal cost (e.g., free program) while interventionists included program cost and stakeholders prioritized CS and program costs.

## Discussion

Prior research suggests that rural CSs are more likely to report lower physical functioning, poor health and physical inactivity compared to their urban counterparts [27, 49]. To assist CSs in coping with these challenges through regular exercise, multilevel input regarding exercise intervention implementation is vital. Results from NGT meetings indicated that each participant type had variable yet overlapping perspectives on factors potentially influencing implementation of a multicomponent exercise intervention for women CSs in rural areas. All five of the major CFIR domains were represented by the responses generated. Potential implementation facilitators related to the “Process” domain were more relevant for stakeholders (29.5%) and interventionists (28.1%), while those related to “Intervention design quality and packaging” of the CFIR Innovation Characteristics domain were of greater relevance to CSs (29.2%) and stakeholders (23.2%). Reponses related to “Patient needs and resources” were more relevant for interventionists (9.4%) and CSs (8.3%), and those related to “Available resources” were more relevant for interventionists (18.8%) and stakeholders (13.7%) (Fig. 1). There were several participant-specific CFIR constructs worth noting, e.g., “Culture” of the Inner Setting domain and “Individual stage of change,” “self-efficacy,” and “Other personal attributes” of the Characteristics of Individuals domain were only valued by CSs. “Tension for change” of Inner Setting domain and “Innovation participants” and “Key stakeholder” of the Process domain were unique for interventionists. “Goals and feedback” and “Network and communication” of Inner Setting domain and “Planning” of Process domain were distinct for stakeholders (Fig. 1). These unique participant-specific CFIR constructs provide us a basis to develop tailored strategies to implement the exercise intervention for different participant types.

Previous studies have shown that organizations delivering exercise interventions for rural CSs must be aware of their clients’ needs and resources [27, 50–52]. Hence, our data suggest that organizations planning to implement exercise programs for CSs in rural settings should include strategies that appropriately address “Patient needs and resources.” For example, our data suggest that implementation strategies should provide CS encouragement and support, increase CS commitment while also optimizing safety, access to expertise, and affordability. The implementation facilitators generated and prioritized by CS participants demonstrated that CSs cared most about “Design quality and packaging” and “Available resources.” Because these two constructs were identified as important to interventionists and Stakeholder participants to a lesser degree, raising interventionist and stakeholder awareness of and ability to address these issues important to CS may be needed.

Research on promoting physical activity to rural CSs suggests that interventionists may play a key role in the implementation of exercise interventions and further research is needed to elucidate characteristics of this role (e.g., acting as a link between stakeholders supportive of such programs and CSs receiving the intervention) [53, 54]. Similar to CSs, interventionists prioritized financial support, safety, and exercise expertise but also endorsed setting realistic exercise goals, providing transportation support, and tracking program recipient progress. These unique viewpoints from the potential interventionists showed their concerns about intervention affordability. They thought setting realistic or attainable goals for CSs would help them to maintain exercise and minimize program drop-out because of unrealistic expectations. Stakeholders should consider addressing these issues when designing implementation strategies that facilitate organizational readiness, interventionists’ engagement, and intervention execution.

Stakeholders, a critical part of translating knowledge into action, can create a “user-friendly” environment or a network for CSs by providing different kinds of support for CSs or even interventionists [28]. Community stakeholders prioritized ways to make doing the intervention easier for an organization. Our data suggests that implementation strategies should focus on engaging an appropriate team of “Qualified trainers” and “Dedicated” individuals which also involves physician support. Controlling cost and enhancing communication within the organization and beyond is also key. They also reiterated several important patient needs and resources warranting attention (e.g., convenience, transportation, support). Given the involvement of a clinical care organization in the study, the mention of factors relevant to policy and incentives is not surprising and was unique to the stakeholder group.

With regard to external validity, the identified CFIR constructs in this report are similar to those from a recent study that focused on implementation of exercise interventions for both clinical and community settings [55]. Although their target population was not CSs, the common CFIR constructs across five domains in intervention implementation support our findings. Another recent study conducted by Canadian researchers using CFIR to categorize factors influencing implementation of an exercise program among CSs reported similar constructs to those from our study, e.g., “Cost” in Intervention Characteristics domain, “Knowledge and beliefs” in Characteristics of Individuals domain [56]. The current findings add to a growing body of literature on the importance of applying CFIR in designing strategies for optimizing exercise intervention implementation [55–58]. Furthermore, the identified potential implementation facilitators may be generalizable to others interested in implementing multicomponent exercise interventions in rural individuals and CSs.

Notably, the data reported here also enhance our prior work in this setting. The results from the NGTs are similar to what we have reported from focus groups with interventionists and stakeholders [28]. Of the CFIR constructs identified in NGT and/or focus group data, two were found in the focus group data only (i.e., “Complexity,” “Structural characteristics”) and six were found in the NGT data only (i.e., “External policies and incentives,” “Networks and communications culture,” “Self-efficacy,” “Other personal attributes,” and “Planning”). Hence, the NGT data expand the constructs identified by focus groups while also advancing our understanding of relative priority differences among participant types, differences that were not discernible using our focus group data. These data also reiterate the importance of addressing factors potentially influencing implementation at multiple levels (e.g., individuals, organizational, community) and generates hypotheses related to the potential importance of appropriately matching implementation strategies to their targeted individuals. Although this is considered an important element of implementation strategy specification and reporting as recommended by Proctor et al. [59], further research is needed to test the value of doing so for these types of interventions in this population.

There are some limitations that should be noted. First, all NGT participants were from a single region of a rural Southeastern US state and the identified factors potentially influencing implementation may not always be generalizable to other regions. However, many are indeed relevant (e.g., affordability, safety, and expertise) based on face value. Second, the majority of CS participants were White and, as such, results from this study might not well represent thoughts of CSs of other races. Third, half of CSs had a history of breast cancer; this might limit the usefulness of the data when implementing with individuals having a history of a cancer other than breast. These limitations are offset by several study strengths. We focused on an underserved and understudied population (i.e., rural women CSs) while also addressing a critically important translational science area (i.e., improving implementation of interventions that increase exercise in a population for whom physical inactivity contributes, in part, to health disparities). Furthermore, we obtained multilevel perspectives on implementation and report novel data regarding the perceived relative importance of factors influencing implementation among different types of intervention “users.” We also used a formally facilitative approach, NGT, to identify potential users’ perspectives. Each NGT meeting was structured to promote equal involvement of all participants and minimize the process loss that arises from various non-task related interpersonal dynamics including extraneous and evaluative discussion that is often encountered in traditional focus groups and unstructured discussion sessions [48]. Hence, we gain a better understanding of how to potentially optimize implementation of exercise interventions for rural CSs based on our data.

## Conclusions and implications

Diverse factors potentially influencing implementation success for which implementation strategies could be developed and tested were identified. Such strategies could potentially influence the implementation of exercise programs for rural CSs, thus reducing an important health disparity. Clearly, the intervention design, cost, patient needs, available resources, and engagement are important across the stakeholder levels. However, the variability in the relative importance of the factors across the three levels supports directing strategies at the level for which it is most desired (e.g., stakeholders are more aware of external policies and regulations, interventionists emphasized resources for readiness, and CSs emphasized intervention design and packaging). The identified and prioritized factors anticipated to influence implementation also reinforce the importance of multilevel implementation strategies for increasing exercise in an underserved, at-risk population. It may be important for individuals involved in planning implementation strategies for exercise interventions to consider how different types of intervention users conceptualize and perceive the importance of CFIR constructs (i.e., strategy targets). Similarly, examining different stakeholders’ perspectives can be used to design for dissemination taking account each participant type’s perspective. Implementation toolkits for exercise programs for rural women CSs should include materials and guidance for addressing the identified factors potentially influencing implementation success. Further research is needed to determine optimal implementation strategies for addressing these factors and the mechanisms by which such strategies influence implementation outcomes through changes in CFIR constructs.

## Data Availability

The dataset analyzed during the current study is not publicly available due to privacy concerns but may be available from the corresponding author on reasonable request.

## Abbreviations

CS: Cancer survivor
CFIR: Consolidated Framework for Implementation Research
NGT: Nominal group technique
